# Global research dynamics in urea cycle disorders: a bibliometric study highlighting key players and future directions

**DOI:** 10.1186/s13023-025-03625-3

**Published:** 2025-03-04

**Authors:** Yan Wang, Xueer Wang, Huiqin Zhang, Binhui Zhu

**Affiliations:** Department of Pediatrics, Xijing Hospital, The Fourth Military Medical University, Xi’an, 710032 China

**Keywords:** Urea cycle disorder, Inborn errors of metabolism, Bibliometric analysis, CiteSpace, VOSviewer

## Abstract

**Background:**

This study aims to explore the research hotspots and trends of urea cycle disorders through bibliometric analysis.

**Methods:**

Using the Web of Science Core Collection as the database, we retrieved literature published from 2007 to 2024. We utilized CiteSpace, VOSviewer, and Bibliometrix R package to conduct a bibliometric visualization analysis, including the number of publications, citation frequency, publishing countries, institutions, journals, authors, references, and keywords.

**Results:**

A total of 926 publications on UCDs were published in 318 journals by 4807 authors at 1494 institutions from 49 countries/regions. The USA had the highest number of publications and citation frequency. The Children’s National Health System in the USA published the most literature. The most frequent collaboration was between the USA and Germany. The journal with the most publications was Molecular Genetics and Metabolism. The author with the most publications was Johannes Häberle. The most frequently cited reference was the 2019 publication of the revised guidelines for the diagnosis and management of UCDs. The identified future research hotspots are expected to focus on “gene therapy”, “mutations” and “efficacy”.

**Conclusion:**

This study is the first bibliometric analysis of publications in the field of UCDs. These findings suggest that European and American countries dominate UCD research, it is necessary to further strengthen global cooperation in the field of UCDs. Early detection of the disease and emerging therapies, including gene therapy, are likely to be future research hotspots.

## Background

The urea cycle is a metabolic pathway that disposes of waste nitrogen by converting ammonia into urea. In 1932, German researchers Hans Krebs and Kurt Henseleit first described the biochemical process of urea production in animals [1]. In 1952, this process was officially termed the “urea cycle.” The first reported urea cycle deficiency, involving argininosuccinate lyase (ASL), was identified in 1958 [2]. Since then, other urea cycle disorders (UCDs) have been identified. Currently, eight subtypes of UCDs are recognized: argininosuccinate lyase deficiency (ASLD), ornithine transcarbamylase deficiency (OTCD), N-acetylglutamate synthase deficiency (NAGSD), carbamoylphosphate synthetase 1 deficiency (CPS1D), argininosuccinate synthetase deficiency (ASSD), arginase 1 deficiency (ARG1D), hyperornithinaemia-hyperammonaemia-homocitrullinuria syndrome (HHHS), and citrin deficiency (Citrin D) [3]. OTCD is the only X-linked urea cycle disorder and is the most common enzymatic deficiency in this pathway, whereas the other UCDs are autosomal recessive genetic disorders [4]. The incidence rates of UCD subtypes vary, with the overall incidence estimated at approximately 1 in 35,000 live births. However, the true incidence may be greater due to underdiagnosis and misdiagnosis [5]. Prenatal DNA analysis is currently used to screen for UCDs, whereas tandem mass spectrometry can detect UCDs and other genetic metabolic disorders in newborns. Treatment for UCDs has evolved from dietary control, medication, and liver transplantation to include emerging therapies such as mRNA and enzyme replacement therapy [6–7]. UCDs often have an insidious onset, with symptoms not always being obvious. UCDs are characterized by high morbidity, significant disability, and a substantial global disease burden.

Bibliometrics is the analysis of publications using statistics to describe or display relationships between published works [8]. Compared to systematic reviews, bibliometrics adopts a quantitative approach to comprehensively analyze a large volume of literature. It not only focuses on a specific area of research but also enables the analysis of research dynamics and trends across an entire field. And bibliometrics has gradually evolved into a branch of information science through continuous innovation and expanded applications. Its core involves the application of mathematical and statistical methods to quantitatively analyze the citation and publication of scientific literature, identifying influential countries, journals, institutions, authors, and frequently cited publications, references, and keywords. Additionally, bibliometrics generates visual representations, such as collaboration networks between countries, institutions, and authors, allowing researchers to understand the developmental history and frontiers of a specific field [9]. Currently, commonly used software for bibliometrics includes the Bibliometrix R package, VOSviewer, and CiteSpace. This study aims to provide a comprehensive review of UCD research through bibliometric visualization methods to reveal the current research status and emerging trends.

## Methodology

### Data sources and search strategies

**Fig. 1:**
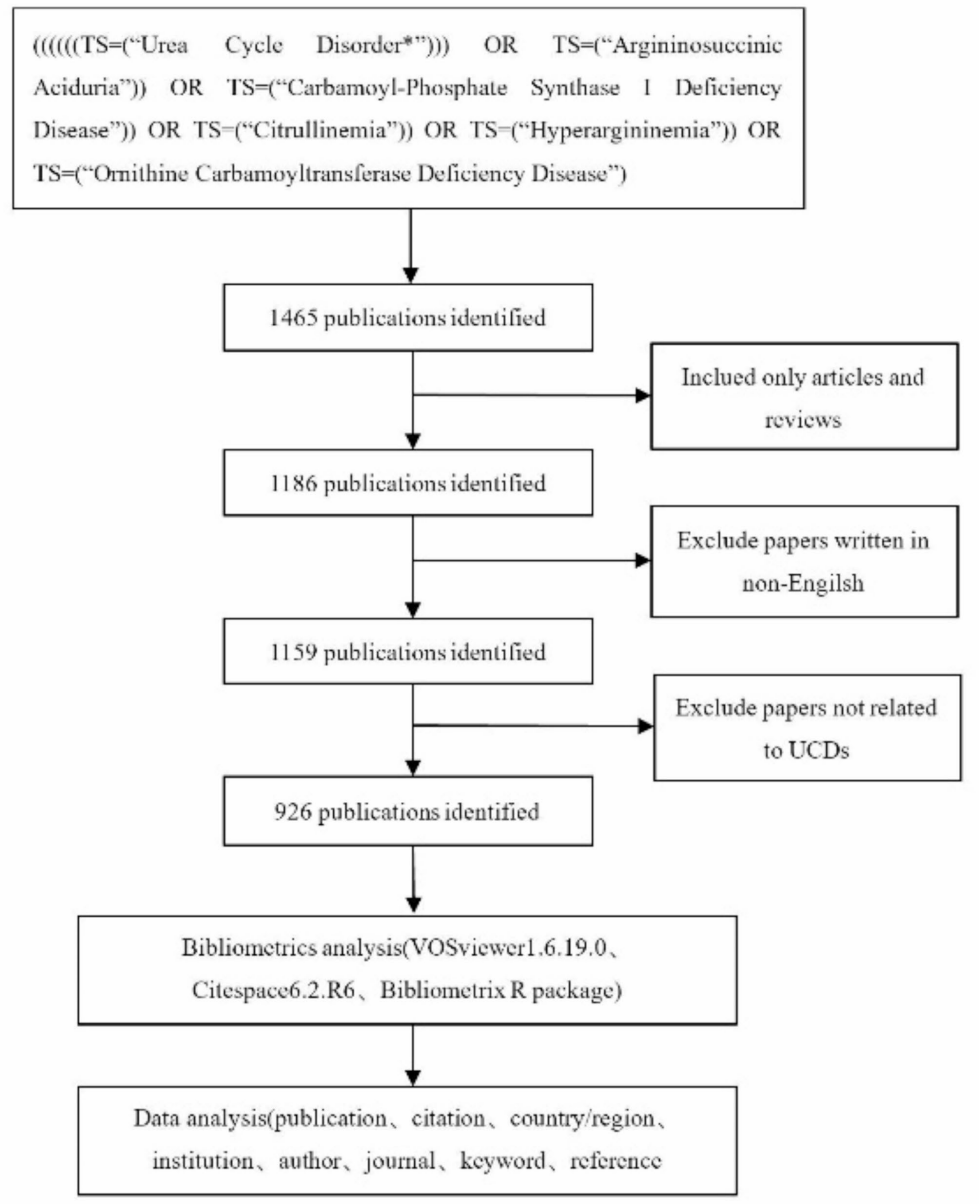
Flowchart of the literature search and screening

The Web of Science Core Collection (WoSCC) is a vital database for accessing global academic information. It encompasses highly authoritative and influential international academic journals, ensuring the reliability and quality of the included literature. As such, it is widely recognized as the most suitable database for bibliometric analysis [10–11]. We accessed the WoSCC database via the Air Force Medical University Library website. To avoid statistical bias caused by database updates, all data collection was completed on July 11, 2024. The search utilized the following terms: “urea cycle disorder”, “argininosuccinic aciduria”, “carbamoyl-phosphate synthase I deficiency disease”, “citrullinemia”, “hyperargininemia”, and “ornithine carbamoyltransferase deficiency disease”. The language was limited to English, and the article type was limited to articles or reviews. Two investigators reached a consensus on the search terms and independently screened the articles by reviewing their abstracts or full texts, including all publications related to UCDs. In cases of disagreement, a third researcher was consulted to make the final decision. The detailed search flowchart is presented in Fig. 1.

### Data extraction and analysis

The included publications were subjected to bibliometric analysis using Microsoft Office Excel 2019, VOSviewer 1.6.19.0, CiteSpace 6.2.R6, and the Bibliometrix R package. We collected and organized data on authors, countries, institutions, publication years, journals, keywords, and citation frequency. Specifically, Excel was used to analyze publication volume, citation frequency, and citation index; Bibliometrix R package was used to analyze journals and national collaboration networks; VOSviewer was used to analyze keywords; and CiteSpace was used to analyze co-cited references.

To determine whether the publication time is associated with its impact, we evaluated the correlation between publication year and citations by calculating the Pearson correlation coefficient. Although citation frequency can be used to assess the impact of a publication, the proximity of the publication date may influence citation frequency to some extent [12]. Therefore, we utilized Citation Index to minimize the effect of publication date on citation frequency, the higher Citation Index value, the more substantial the impact of the literature. The calculation method is as follows [13]: $\:\text{C}\text{i}\text{t}\text{a}\text{t}\text{i}\text{o}\text{n}\:\text{I}\text{n}\text{d}\text{e}\text{x}=\frac{\text{T}\text{o}\text{t}\text{a}\text{l}\:\text{C}\text{i}\text{t}\text{a}\text{t}\text{i}\text{o}\text{n}\:\text{F}\text{r}\text{e}\text{q}\text{u}\text{e}\text{n}\text{c}\text{y}}{\text{P}\text{u}\text{b}\text{l}\text{i}\text{c}\text{a}\text{t}\text{i}\text{o}\text{n}\:\text{Y}\text{e}\text{a}\text{r}\text{s}.}$

## Results

### General information

A total of 926 articles were ultimately included in the study. These articles were published from 2007 to 2024, with an average citation frequency of 20.2, involving 49 countries, 1494 research institutions, 318 journals, 4807 authors, and 18,905 references.

### Annual publications and citation index

**Fig. 2:**
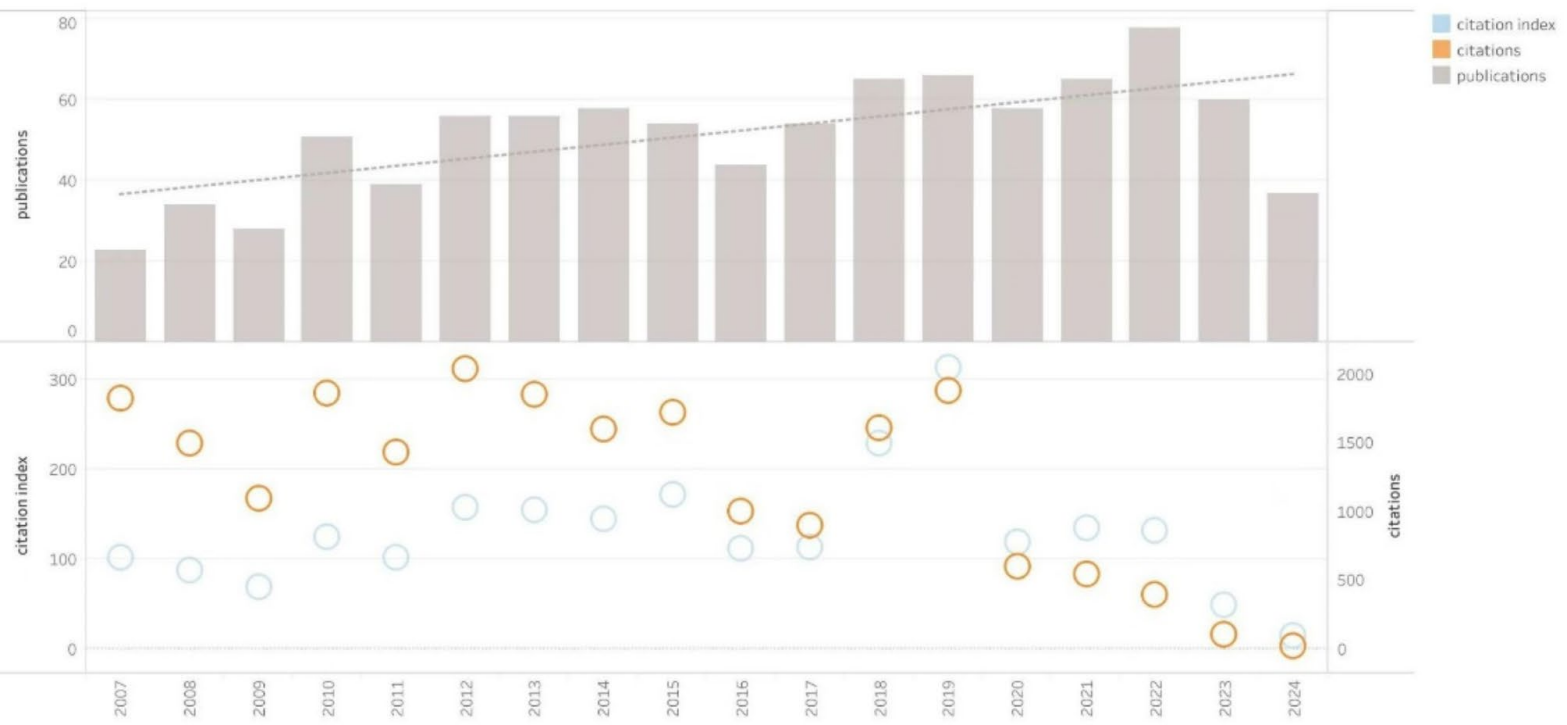
Annual publications, citations and citation index of UCDs from 2007–2024

Figure 2 shows the annual publications, citation frequency, and citation index of UCDs. In Fig. 2, the bar chart represents the annual publication volume, and the dashed gray line indicates the linear trend line for the annual publication volume. It can be observed that since 2007, the annual number of publications in the UCD field has shown a growing trend. In Fig. 2, the yellow circles represent citation frequency, and the blue circles represent the citation index. The top publications occurred in 2022, with 78 articles; the highest citation frequency was in 2012, with 2,036 citations; and the highest citation index was in 2019, with a value of 313.

We analyzed the correlation between publication year and citation frequency, yielding the Pearson correlation coefficient was − 0.736 (*p* = 0.001), indicating a significant negative correlation: the earlier the publication year, the higher the citation frequency. In the analysis of the correlation between publication year and citation index, the Pearson correlation coefficient was 0.027 (*p* = 0.915), indicating no correlation between publication year and citation index, this indicates that the citation index can indeed reduce the impact of publication time on citation frequency. Therefore, we calculated the citation index for each article, and the article with the highest citation index was “Branched-chain amino acids in health and disease: metabolism, alterations in blood plasma, and as supplements”, published in 2018 [14]. This article delves into the importance of branched-chain amino acids (BCAAs) in both health maintenance and disease progression. Its metabolic pathways, alterations within the blood plasma, and application as nutritional supplements have been explored. The article mentioned that branched-chain amino acids can detoxify ammonia into glutamine, thereby alleviating hyperammonaemia; additionally, the use of nitrogen-scavenging compounds (such as sodium phenylbutyrate) can lead to a decrease in plasma branched-chain amino acid levels. Consequently, certain patients with UCDs opt to supplement with branched-chain amino acids as part of their treatment regimen, as this can help manage symptoms and enhance their overall condition more effectively.

### Countries and institutions

**Fig. 3:**
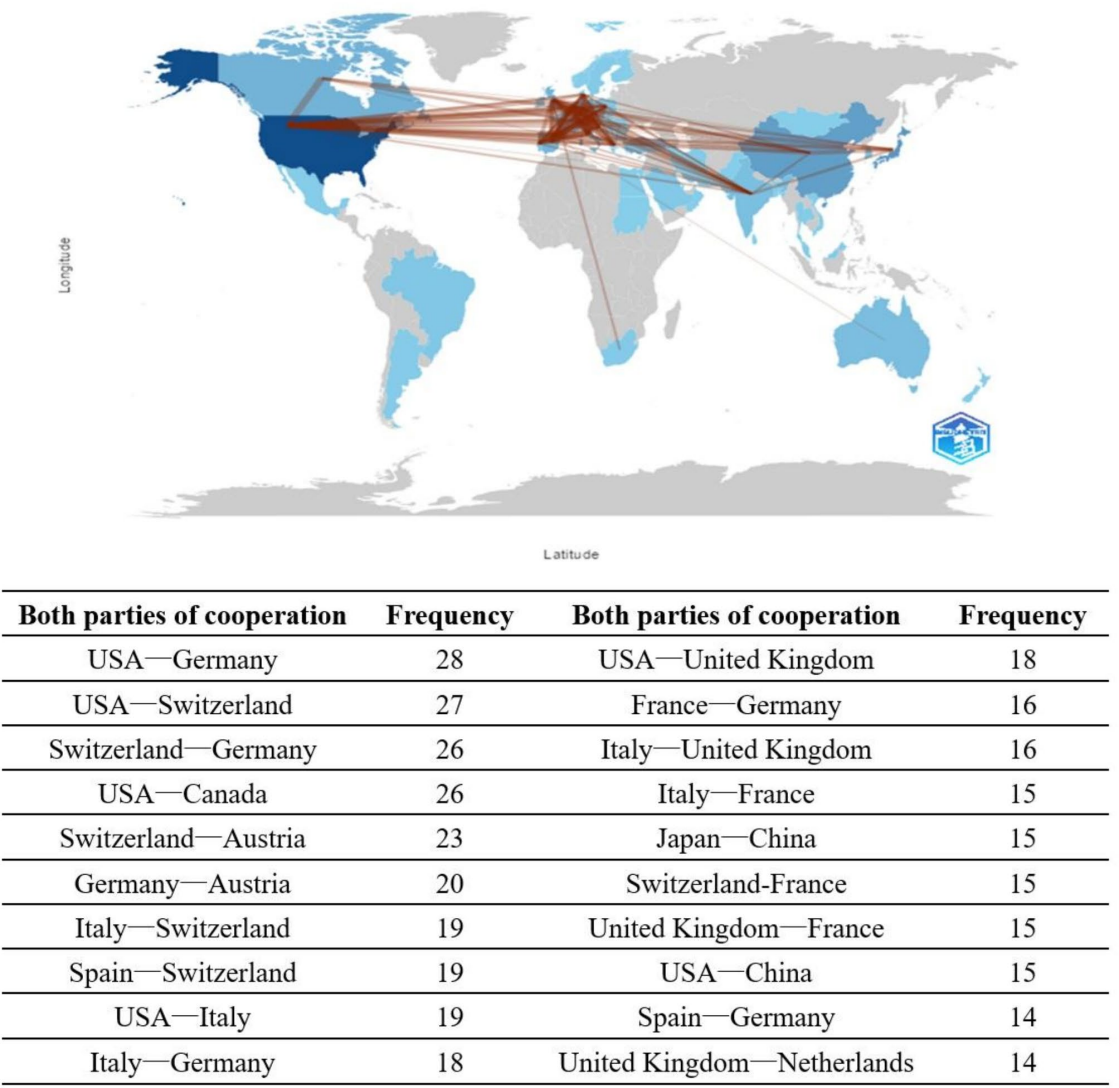
Geographic distribution map of different countries in the UCD field

The top 10 countries with the most publications and the highest total citation frequencies are shown in Table 1. The United States ranked first with 259 articles, followed by Japan, China, Italy, and Germany. The five countries ranking highest in total citation frequency are the United States, Japan, Germany, Switzerland, and China. Figure 3 depicts the national collaboration network, where denser connections indicate more frequent collaborations between countries. Overall, the USA and European countries, as well as European countries among themselves, show closer collaboration. Among these, the USA and Germany, the USA and Switzerland, and Germany and Switzerland exhibit the most frequent collaborations. In Asia, China and Japan collaborate relatively frequently, with a collaboration frequency of 15 occurrences. Table 2 summarizes the top 10 research institutions with the most publications, accounting for 55.4% of the included literature. Among them, the National Children’s Medical Center of the United States has the most publications; the University Children’s Hospital Zurich and Children’s Research Centre ranks second in terms of publications, and this institution also has the highest centrality, indicating its significant influence in the field of UCD research.

**Table 1:** The top 10 countries contributing to Urea cycle disorder research

Country	Number of Publications	Country	Number of Citations
USA	259	USA	6635
Japan	103	Japan	1701
China	93	Germany	1408
Italy	51	Switzerland	1256
Germany	43	China	1022
UK	39	UK	880
Switzerland	33	Canada	788
Turkey	30	Italy	773
Spain	28	Australia	685
Canada	27	Czech	444

**Table 2:** The top 10 productive institutions contributing to Urea cycle disorder research

Institutions	Location	Number of publications	Centrality
Children’s National Health System	USA	97	0.08
University Children’s Hospital Zurich	Switzerland	80	0.23
Baylor College of Medicine	USA	62	0.08
University of California System	USA	48	0.04
Ruprecht Karls University Heidelberg	Germany	46	0.1
George Washington University	USA	42	0.01
University of California Los Angeles	USA	36	0.01
CIBER - Centro de Investigacion Biomedica en Red	Spain	35	0.09
Kumamoto University	Japan	34	0.05
Assistance Publique Hopitaux Paris (APHP)	France	33	0.17

### Analysis of journals and authors

Table 3 lists the top 10 journals that have reported research findings in the fields of endocrinology and metabolism, molecular genetics, and rare diseases. Table 4 shows the top 10 most productive authors who contributed to UCDs, whose publication numbers account for 42.2% of the total. Among them, Johannes Häberle from Switzerland ranks first in terms of the number of publications, citations, and H-index.

**Table 3:** The top 10 most productive journals for Urea cycle disorder research

Journal	Number of publications	IF(2023)	JCR Quartile (2023)	H Index
Molecular Genetics and Metabolism	108	3.7	Q2	32
Journal of Inherited Metabolic Disease	83	4.2	Q1	27
Molecular Genetics and Metabolism Reports	33	1.8	Q3	8
Orphanet Journal of Rare Diseases	25	3.4	Q2	14
Pediatric Transplantation	16	1.2	Q3	10
Clinica Chimica Acta	14	3.2	Q2	9
Human Mutation	11	3.3	Q2	10
Pediatric Neurology	11	3.2	Q2	8
Gene	10	2.6	Q2	6
Nutrients	10	4.8	Q1	4

**Table 4:** The top 10 most productive authors who contributed to Urea cycle disorder research

Author	Country	Publications	Citations	H Index
Johannes Häberle	Switzerland	71	2423	26
Keiko Kobayashi	Japan	31	1000	20
Takeyori Saheki	Japan	31	967	19
George A Diaz	USA	29	835	16
Stefan Kölker	Germany	29	855	16
Georg F Hoffmann	Germany	27	784	16
Sandesh C S Nagamani	USA	26	693	15
Carlo Dionisi-Vici	Italy	25	1430	16
Brendan H Lee	USA	25	1095	18
Uta Lichter-Konecki	USA	25	839	17

### Keyword analysis

**Fig. 4:**
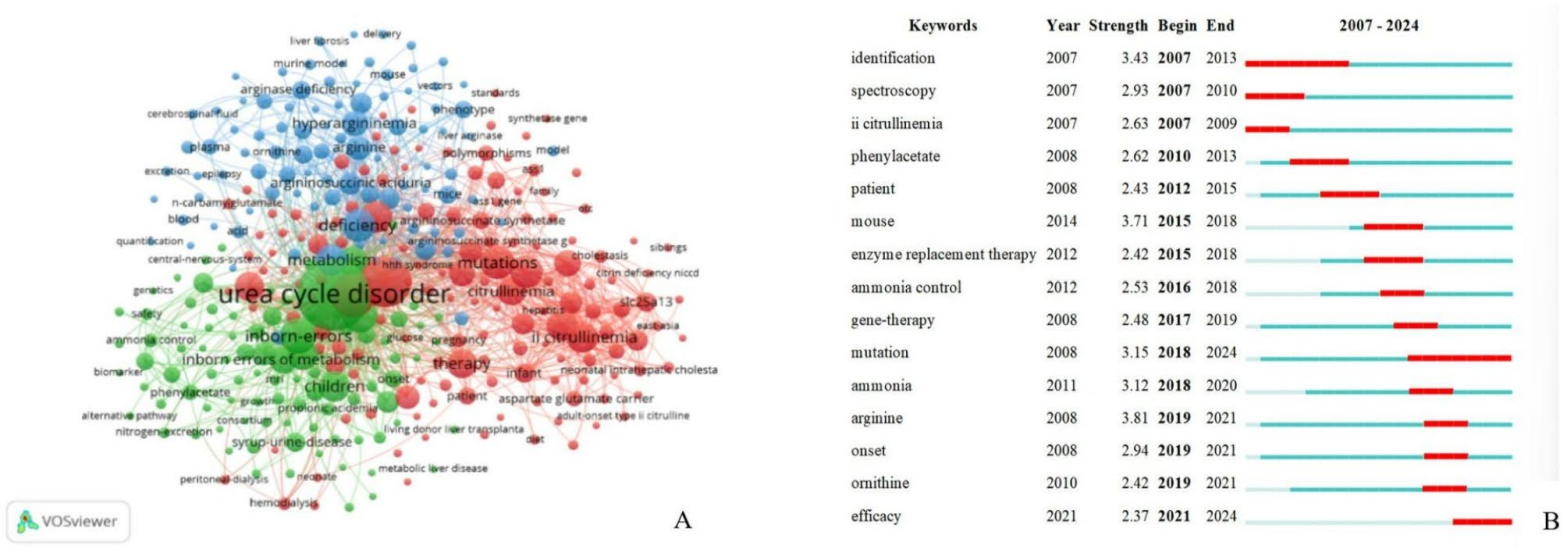
(**A**) Network visualization of the co-occurrence keywords. (**B**) The top 15 keywords with the strongest citation bursts of publications in the field of UCDs from 2007–2024

Using VOSviewer software, we analyzed the keywords, in the generated network, the size of the spheres represents the frequency of keyword occurrences in the publications. The lines connecting keywords indicate their co-occurrence in the same publication. The position of keywords on the map reflects their relative relevance, while the color scheme represents the clustering patterns identified in this study [15]. As shown in Fig. 4A, there are three key areas in the UCDs. The first area is the blue cluster, which mainly involves clinical research on UCDs, including disease subtypes, clinical characteristics and complications, such as hyperammonaemia, OTCD, protein, gene, and encephalopathy. The most common keyword in this area is “hyperammonaemia”. The second area, indicated by the green cluster, involves the diagnosis, treatment, and management of UCDs, including tandem mass spectrometry, liver transplantation, sodium phenylbutyrate, management, survival, etc. The third area, represented by the red cluster, encompasses basic research on UCDs, including metabolic pathways, mouse models, gene expression, gene therapy, etc. The most common keyword in this area is “metabolism”. Although the three areas have different focuses, the disease name as a keyword overlaps in each area, such as OTCD and ASS. Figure 4B shows the 15 keywords with the strongest citation bursts in the UCD field, among which the strongest burst keyword is “arginine” (Strength = 3.81). “Identification” (2007–2013) and “mutation” (2018–2024) had the longest burst durations. The keyword “efficacy” still bursts. Moreover, keywords that burst early, such as “identification,” “spectroscopy,” and “II citrullinemia,” focus on the clinical characteristics and diagnosis of UCDs. While keywords such as “gene therapy, mutation” have burst in recent years, they are increasingly centered on the genetic characteristics and emerging therapies of UCDs.

### Co-cited references and references burst

**Fig. 5:**
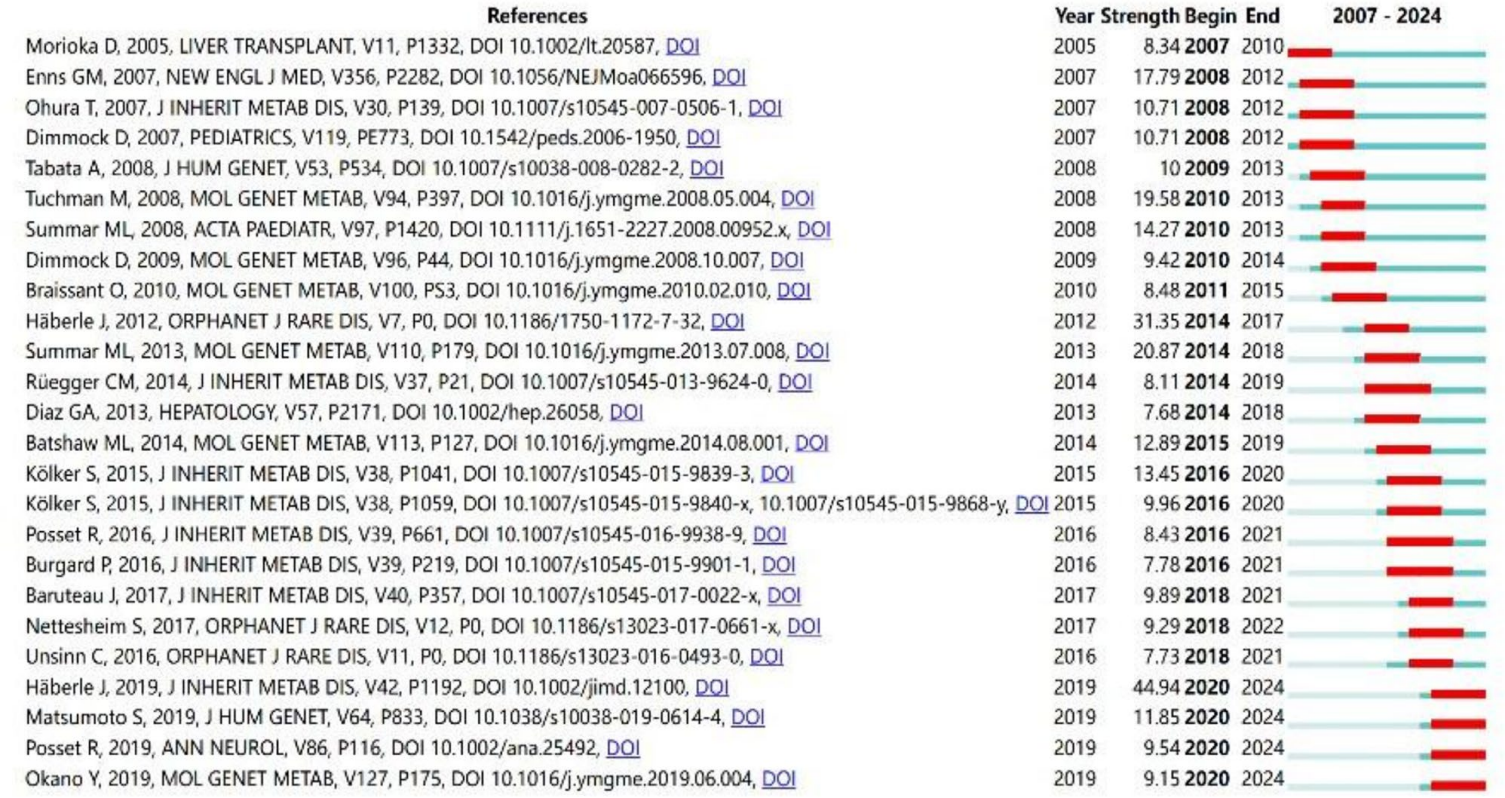
The top 25 references with the strongest citation bursts

Co-cited reference analysis was performed via CiteSpace software. Table 5 summarizes the top 10 co-cited references in the UCD field published between 2007 and 2024, including UCD diagnosis and management guidelines, sodium phenylbutyrate and sodium benzoate treatment of UCDs, etc. The reference with the most cocitations is “Suggested guidelines for the diagnosis and management of urea cycle disorders: First revision”, which is a content revision of the UCD guidelines published in 2012, published by Johannes Häberle in 2019 [16]. Figure 5 shows the top 25 references with the strongest citation bursts; the reference with the strongest burst (strength = 44.94) is still the UCD 2019 guideline mentioned above, and the burst continues until 2024. Likewise, three more publications from 2019 have retained a sustained momentum of investigation until 2024. These three publications encompass the following topics: the study of the causes, diagnosis, and management of UCDs; the enhancement of cognitive functions through the treatment of UCDs; and preventive measures aimed at mitigating the risk of citrullinemia.

**Table 5:** The top 10 cited references for Urea cycle disorder research

	Title	Year	Author	Journal	Number of Co-citations	Strength
1	Suggested guidelines for the diagnosis and management of urea cycle disorders: First revision	2019	Johannes Häberle	J Inherit Metab Dis	114	44.49
2	Suggested guidelines for the diagnosis and management of urea cycle disorders	2012	Johannes Häberle	Orphanet J Rare Dis	74	31.35
3	The incidence of urea cycle disorders	2013	Marshall L Summar	Mol Genet Metab	51	20.87
4	Cross-sectional multicenter study of patients with urea cycle disorders in the United States	2008	Mendel Tuchman	Mol Genet Metab	41	19.58
5	Survival after treatment with phenylacetate and benzoate for urea-cycle disorders	2007	Gregory M Enns	N Engl J Med	38	17.79
6	The phenotypic spectrum of organic acidurias and urea cycle disorders. Part 1: the initial presentation	2015	Stefan Kölker	J Inherit Metab Dis	35	13.45
7	A longitudinal study of urea cycle disorders	2014	Mark L Batshaw	Mol Genet Metab	33	12.89
8	Urea cycle disorders-update	2019	Shirou Matsumoto	J Hum Genet	31	11.85
9	Diagnosis, symptoms, frequency and mortality of 260 patients with urea cycle disorders from a 21-year, multicentre study of acute hyperammonaemic episodes	2008	Marshall L Summar	Acta Paediatr	30	14.27
10	Incidence, disease onset and short-term outcome in urea cycle disorders -cross-border surveillance in Germany, Austria and Switzerland	2017	Susanne Nettesheim	Orphanet J Rare Dis	27	9.29

## Discussion

This study clearly demonstrates that, starting in 2007, the annual number of published papers and citations in the UCD field has consistently grown, reaching its highest point in 2022. There is no significant correlation between the citation index and the publication year, and using the publication year to adjust the citation index may help in evaluating the impact of papers. The highest citation index article in this study is a review published in 2018 on the role and clinical applications of branched-chain amino acids, and the article also shows potential applications for conditions such as chronic kidney failure and diabetes [14]. Compared with articles that describe only rare diseases such as UCD, this review covers a broader range of diseases, which may be a significant factor contributing to its high citation index. Through bibliometric visualization analysis of the UCD field literature, the development trends of UCD research have been comprehensively outlined, and future research hotspots have been predicted. As of 11 July 2024, 926 publications had been published in 318 journals by 4807 authors from 1494 institutions in 49 countries/regions.

According to the analysis of publication countries, the United States ranks first in both total number of publications and total citation frequency, indicating that the U.S. is a high-output and leading country in the UCD research field. It also frequently collaborates with other countries, occupying 3 out of the 5 most cooperative country groups. Japan ranks second in both indicators; its number of publications is less than half of that of the U.S. (103/259), and the total number of citations is only 25.6% of that of the U.S. (1701/6635), revealing a significant gap in both quantity and quality compared with those of the U.S. China ranks third in the number of publications but fifth in total citation frequency, indicating an imbalance between quantity and quality. This issue can be addressed from the following aspects: (1) No Asian country is among the top 10 in terms of cooperation in the UCD field; strengthening cooperation with European and American countries could lead to broader data resource sharing and expand the research scope. (2) Closely paying attention to technological innovation in this field can enhance publication quality and deepen research. Among the top 10 institutions in terms of the number of publications, half are from the U.S. This explains why the U.S. is the most productive country in the UCD field. These findings indicate an imbalance in global academic resources within UCD research, and establishing world-class academic institutions is an essential foundation for improving national academic status. From the analysis of global collaboration patterns, cooperation in the UCD field is dominated by partnerships between European and American countries. This can be attributed to the fact that UCD is a rare disease with a low incidence rate, and its research spans multiple fields, including genetics, metabolic diseases, clinical medicine, and drug development. European and American countries hold significant advantages in these areas. Firstly, these regions benefit from stronger research funding in biomedical and healthcare fields. Institutions such as the National Institutes of Health (NIH) in the United States, the European Molecular Biology Laboratory (EMBL), and the Swiss National Science Foundation (SNSF) have provided substantial financial support for both basic and clinical research on UCD [17–20]. Secondly, in the development of drugs for UCD, policies such as the U.S. Orphan Drug Act, the European Union (EU), and the European Medicines Agency (EMA) provide incentives for pharmaceutical companies through funding support, tax breaks, and market exclusivity, thereby accelerating the development of new drugs. This explains why pharmaceutical companies in the UCD field are more inclined to collaborate with European and American countries that have favorable policies, further promoting cooperation between these regions [21–22]. Additionally, the widespread use of English as an official language in most European and American countries creates favorable conditions for collaboration. In this study, we found that, in addition to European and American countries, Japan and China also have a considerable number of publications in the UCD field. Looking ahead, we hope to foster global collaboration in this field through diverse funding channels such as international funds, government grants, patient organizations, and private foundations. This would help avoid biases in drug treatment safety and efficacy caused by factors such as ethnicity and geography, ultimately striving to provide patients with better treatment options and improved quality of life [23].

Among the top 10 most productive authors, Johannes Häberle, a scholar from the University Children’s Hospital of Zurich, ranks highest in terms of publication count, citation frequency, and H-index in the UCD field. He has published 71 related articles, which have been cited 2,423 times. As the first author, he published the 2012 and 2019 versions of the guidelines for the diagnosis and treatment of UCD [16, 24]. Additionally, he has published multiple high-quality research articles and reviews in international authoritative journals such as the *New England Journal of Medicine*,* Nature Medicine*, and *Lancet Diabetes & Endocrinology* [25–27]. The Japanese scholar Saheki Takeyori ranks second, with a focus on Citrin deficiency [28–31].

Among the top 10 journals by publication volume, *Molecular Genetics and Metabolism* (IF = 3.7), *Journal of Inherited Metabolic Disease* (IF = 4.3), and *Molecular Genetics and Metabolism Reports* (IF = 1.9) are the most productive journals. These journals specialize in the field of genetic metabolic diseases and mainly publish research related to genetics and metabolic disorders, including basic research, clinical studies, and translational medicine. They focus on disease screening and diagnosis, clinical management and treatment, and exploration of the pathogenic mechanisms of genetic metabolic diseases. Their target audience includes experts in molecular genetic metabolic diseases, specialized clinicians, researchers, and scholars in related fields. The lower IF may be due to the smaller number of professionals researching these rare diseases. Therefore, a relatively low IF does not fully reflect the scientific status of these journals in the field of metabolic disease research. In the future, these journals are expected to publish more important research results, and the IF and scientific value will increase. Focusing on these journals can help researchers update the latest developments in UCD research and assist in quickly identifying suitable journals for submission, thus avoiding delays in the research process.

Keywords are summaries of the core ideas of an article and are often considered important indicators reflecting the research directions and hotspots in a specific field. The author used VOSviewer to analyze the keywords of 926 UCD-related studies and divided the research field into three aspects through cluster visualization analysis: clinical research, diagnosis and management, and basic research. Clinical research has focused mainly on the clinical subtypes of diseases and their characteristics. UCDs are a group of inherited metabolic diseases caused by defects in enzymes and transport proteins involved in the urea cycle, leading to impaired amino acid metabolism. As a result, the ammonia produced cannot be excreted through the urea cycle, causing elevated blood ammonia levels. With the gradual understanding of the disease and progress of detection technology, methods such as tandem mass spectrometry and genetic testing have gradually been applied in clinical practice, making it possible to distinguish UCD from other genetic metabolic diseases that can present with hyperammonaemia, such as organic acidaemia (e.g., methylmalonic acidaemia, propionic acidaemia, etc.), fatty acid oxidation disorders (e.g., medium-chain acyl-CoA dehydrogenase deficiency, very long-chain acyl-CoA dehydrogenase deficiency, etc.), and amino acid metabolic diseases (e.g., lysinuric protein intolerance, maple syrup urine disease, etc.), and gradually, the cessation of the intake of natural proteins can reduce the production of ammonia in the body. Oral medications, including nitrogen scavengers (sodium benzoate, sodium phenylbutyrate, and phenylbutyrate glycerol ester), urea cycle activators/substrate supplements (N-carbamylglutamate, arginine, and citrulline), and other auxiliary drugs (lactulose and L-carnitine), can be used to control UCD [32–34]. However, liver transplantation remains the only curative option at present [35–36]. With the ongoing progress in basic research, researchers have gradually gained a deeper understanding of the pathogenic mechanisms of UCD. Since the 1990s, emerging therapies such as gene therapy have attracted the attention of the public and are now in a stage of clinical translation [37]. Ultragenyx-sponsored DTX301 (an investigational AAV-based gene therapy) packages the optimized OTC gene into an adenovirus vector. After a single peripheral intravenous infusion, long-lasting hepatic gene expression occurs. In phase I/II clinical trials (NCT02991144) involving adults, the study consisted of four cohorts. The first three cohorts, each comprising three patients, received intravenous infusions of scAAV8-OTC at doses of 2.0 × 10¹² GC/kg, 6.0 × 10¹² GC/kg, and 1.0 × 10¹³ GC/kg, respectively. In the fourth cohort, two patients were treated with 1 × 10¹³ GC/kg of scAAV8-OTC combined with prophylactic corticosteroids. Among the 11 treated patients, 7 responded positively to the treatment, with stable clinical manifestations and metabolic states. No serious adverse events, infusion-related reactions, or dose-limiting toxicities associated with the therapy were reported across all dose groups [38]. This treatment method has now entered phase III clinical trials (NCT05345171) [39], which is a randomized, double-blind, placebo-controlled study of DTX301. The study targets late-onset OTCD patients aged 12 years and older. Participants will be randomly assigned in a 1:1 ratio to either the DTX301 group or the placebo group, with close follow-up for 64 weeks. The objective of the study is to evaluate whether treatment with DTX301 can safely maintain blood ammonia levels without protein intake restrictions and cessation of related supplemental medications. The study is scheduled to be completed in December 2028, and no related results have been published yet. Another clinical trial sponsored by University College London is currently recruiting patients (NCT05092685) to treat OTCD in children under 16 years of age, using the hepatotropic capsid AAV-LK03 to transduce human liver cells for enhanced efficacy [40]. On April 4, 2024, the FDA approved the clinical study of ECUR-506 for the treatment of OTCD patients under 9 months of age (including newborns). ECUR-506 consists of two AAV vectors, one delivering the ARCUS nuclease and the other delivering the required functional OTC gene. Through gene-editing techniques, the OTC gene-carrying therapy is administered via intravenous infusion. On May 7, 2024, it was announced that ECUR-506 had received FDA fast track certification. Currently, it has entered the recruitment phase for phase I/II clinical trials (NCT06255782), with plans to enroll 13 participants [41], recruitment is taking place at four hospitals in the United States and the United Kingdom: UCLA Mattel Children’s Hospital, Children’s Hospital of Colorado - Anschutz Medical Campus, Great Ormond Street Hospital, and The Newcastle upon Tyne Hospitals NHS Foundation Trust - Great North Children’s Hospital. The first three institutions have already begun recruitment, while the last one has yet to start officially. Early-onset OTCD is a critical condition with high mortality rates, and survivors often experience varying degrees of neurological sequelae. Early treatment is key to reducing mortality and disability rates in these patients. This therapy marks the first time a gene therapy for urea cycle disorders has extended its indication to include newborns, offering a new avenue of treatment and hope for children with early-onset OTCD. The clinical trials NCT05345171, NCT05092685, and NCT06255782 are all in the recruitment phase, with no results published yet. We will continue to monitor their progress. As mRNA technology has matured, its safety and effectiveness have been verified globally. This technology can convert the mRNAs of urea cycle-related enzymes into lipid nanoparticles or nonviral dual nanoparticle delivery systems, thereby targeting the defective cells to transiently express the required proteins and alleviate the disease. The mRNA therapy for OTCD (ARCT-810), sponsored by Arcturus Therapeutics, has completed phase Ia and Ib clinical trials and has successfully initiated phase II clinical trials (NCT05526066) [42]. mRNA therapy has also achieved positive results in animal models of ARG1 deficiency and ASL deficiency. In addition, liver-derived mesenchymal stem cell therapy and enzyme replacement therapy involving polyethylene glycol arginase are also undergoing corresponding clinical trials worldwide [43–44]. The future of these treatments is very promising, and scholars can explore further on the basis of these findings.

Co-citation analysis is an effective method for evaluating the correlation level between articles. It is generally believed that the more times an article is cited, the greater its importance in the professional field [45]. The reference co-cited most frequently and with the highest burst intensity is the revised Urea Cycle Disorders Diagnosis and Treatment Guidelines published in 2019. This review article serves as an important clinical decision-making tool to bridge the gap between existing evidence-based medicine and clinical practice. This guideline was revised by a panel of experts, consisting of 17 experts from 9 countries in Europe and Israel, on the basis of the 2012 version, further accumulating data and experience. In addition to deepening the understanding of the clinical characteristics of the disease, it has expanded UCD newborn screening in terms of diagnosis and treatment and added new therapies, such as phenylbutyrate glycerol ester and gene therapy, emphasizing the importance of multidisciplinary teamwork, and recommending lifelong monitoring for all UCD patients. This comprehensive guide has deepened clinicians’ understanding of UCDs, systematically reviewed existing treatments, and provided crucial guidance for future research directions and the development of novel therapies [12]. Its ultimate aim is to better support clinical practice and improve patient care. Furthermore, it has laid a foundation for the development of subsequent consensus guidelines in the UCD field [3, 46].

This study also has certain limitations. First, this study used the WoSCC database purchased by our hospital (with the starting year of the literature being 2007) as the data source and did not include other databases, such as PubMed, Cochrane, Scopus, and Embase, which may have led to the omission of some research results. However, WoSCC covers the majority of high-impact articles indexed in these databases and offers significant advantages compared to other platforms. These advantages include extensive multidisciplinary coverage, advanced search and filtering options, and powerful citation tracking tools, making it particularly suitable for interdisciplinary and bibliometric studies. Additionally, other databases besides WoSCC do not provide exportable reference citation data, which precludes subsequent co-citation reference analyses. High-citation reference analysis, however, provides a unique perspective on core literature, research relationships, and academic networks within the field of UCD research. It also helps uncover research hotspots and developmental trends, making it one of the key advantages of bibliometric analysis. Second, only English publications were included in the search, while non-English publications were excluded, which may lead to the omission of some publications. However, as English is the official language commonly used for international communication, most high-impact articles are published in English. Focusing on English publications ensures the inclusion of high-quality research, minimizes translation challenges and inaccuracies, and enhances the rigor and precision of our analysis by manually screening potentially irrelevant articles. In addition, although three authors were involved in the literature screening process, factors such as differences in knowledge background and personal perspectives might introduce some bias in the screening results. Finally, although manual revisions and standardizations were applied to certain results, including keywords, to reduce bias caused by different expressions of the same concept, the biases introduced by the aforementioned factors cannot be entirely eliminated.

In summary, this study is the first to use visualization software to objectively conduct bibliometric analysis on UCD-related publications. The findings reveal that European and American countries dominate UCD research. In the future, it is necessary to further strengthen global cooperation in the field of UCDs and expand the depth and breadth of research. Early detection of the disease and emerging therapies, including gene therapy, are likely to be future research hotspots.
